# Intermolecular distance measurement with TNT suppressor on the M13 bacteriophage-based Förster resonance energy transfer system

**DOI:** 10.1038/s41598-018-36990-0

**Published:** 2019-01-24

**Authors:** Inhong Kim, Hyerin Song, Chuntae Kim, Minwoo Kim, Kwangseuk Kyhm, Kyujung Kim, Jin-Woo Oh

**Affiliations:** 1 School of Electrical Engineering and Computer Science, Gwangju Institute of Science and Technology, Gwangju, 61005 Republic of Korea; 2 Department of Cogno-Mechatronics Engineering, Pusan National University, Busan, 46241 Republic of Korea; 3 Department of Nano Fusion Technology, Pusan National University, Busan, 46241 Republic of Korea; 4 Department of Nanoenergy Engineering, Pusan National University, Busan, 46241 Republic of Korea

## Abstract

An M13 bacteriophage-based Förster resonance energy transfer (FRET) system is developed to estimate intermolecular distance at the nanoscale using a complex of CdSSe/ZnS nanocrystal quantum dots, genetically engineered M13 bacteriophages labeled with fluorescein isothiocyanate and trinitrotoluene (TNT) as an inhibitor. In the absence of trinitrotoluene, it is observed that a significant spectral shift from blue to green occur, which represents efficient energy transfer through dipole-dipole coupling between donor and acceptor, or FRET-on mode. On the other hand, in the presence of trinitrotoluene, the energy transfer is suppressed, since the donor-to-acceptor intermolecular distance is detuned by the specific capturing of TNT by the M13 bacteriophage, denoted as FRET-off mode. These noble features are confirmed by changes in the fluorescence intensity and the fluorescence decay curve. TNT addition to our system results in reducing the total energy transfer efficiency considerably from 16.1% to 7.6% compared to that in the non-TNT condition, while the exciton decay rate is significantly enhanced. In particular, we confirm that the energy transfer efficiency satisfies the original intermolecular distance dependence of FRET. The relative donor-to-acceptor distance is changed from 70.03 Å to 80.61 Å by inclusion of TNT.

## Introduction

In the biological recognition process, the molecular interaction between receptors and analytes is often associated with a conformational change due to specific physical or chemical binding^1–3^. The optical transduction of such conformational changes for complex molecules provides a method of identifying unknowns, understanding transient molecular dynamics, and devising bio-optical sensing mechanisms^4–8^. Enormous research efforts have been made to optically transduce conformational changes of complex molecules, and these have resulted in the development of various optical sensing techniques, such as fluorescent assays^9–13^, surface plasmon resonance (SPR), localized surface plasmon resonance (LSPR)^14–17^, surface-enhanced Raman scattering (SERS)^18–21^, and Förster resonance energy transfer (FRET)^22–26^. In particular, the FRET technique has been extensively investigated over decades since it offers a broad view of molecular dynamics as a spectroscopic ruler^27^. FRET-based sensing facilitates the visualization of receptor-analyte interactions through the detection of color change and provides clues regarding relative intermolecular distances between reacting molecules through time-integrated or time-resolved analysis. Consequently, the FRET-based approach has been widely utilized in various applications such as medical diagnostics^28^, biomarkers^29,30^, cell imaging^31,32^, DNA sequence analysis^33,34^, molecular interaction in DNA or proteins^35–41^, and chemosensors^25,42,43^.

However, if receptors do not have exceptional affinity and specificity for an analyte, the noticeable advantages of FRET cannot be guaranteed. Recently, unique functions of biomaterials from protein display platform have been utilized in interesting way, expanding their usage for novel applicationsincluding biosensing^44^, cancer therapy^45^, stem cell control^46^ and gene therapy^47^.

Especially, the M13 bacteriophage (phage) has attracted attention as a next-generation receptor material^48–53^ due to its specific binding properties and well-defined shape (cylindrical shape, 880 nm in length and 6.6 nm in diameter)^54,55^ for FRET-based applications^56–58^. By using a site-specified M13 phage, these applications demonstrate excellent spectral changes and rapid fluorescence quenching. The suitability of the M13 phage for FRET-based sensing applications is proven by its structural features. Since the M13 phage is covered with 2,700 copies of a major coat protein (pVIII) on its surface and minor proteins (pIII, pVI, pVII and pIX) at both of its ends, site-specific modification for binding with incoming particles is straightforward^49^. In particular, the M13 phage has outstanding advantages in labelling simplicity and a high sensitivity for analyte detection^50,59^.

Neverthless, the role of the M13 phage as a scaffold for immobilization of fluorescent dyes in a FRET-based optical application is limited. Considering that resonant coupling between dipoles is within 100 Å^60^, the dimensions of the M13 phage are much larger. On this account, the M13 phage is often used in a single-molecular FRET scheme, whereby a donor and acceptor pair is immobilized onto the M13 phage^56–58^. The dipolar interaction in an M13 phage-based FRET system occurs between immobilized dyes on two neighboring N-termini of pVIII proteins. The intermolecular distance between them is about 24~32 Å^56^. This restriction can affect the sensitivity of FRET-based analyte sensing because it limits the number of specific peptides of the M13 phage eligible to participate in receptor-analyte reactions.

In this work, we designed an M13 phage-based FRET system using a complex of water-soluble CdSSe/ZnS nanocrystal quantum dots (donor, blue emission, NQDs), a genetically engineered M13 bacteriophage labeled with fluorescein isothiocyanate (acceptor, green emission) and trinitrotoluene (TNT) as an inhibitor. The novel performance features of the M13 phage-based FRET system were practically confirmed by fluorescence spectra and fluorescence decay curves. Also, we applied the M13 phage-based FRET system to validate the performance of the TNT suppression process in reducing the total energy transfer efficiency. Finally, we estimated the relative intermolecular distance between a donor and acceptor based on the energy transfer efficiency.

## Results

**Figure 1:**
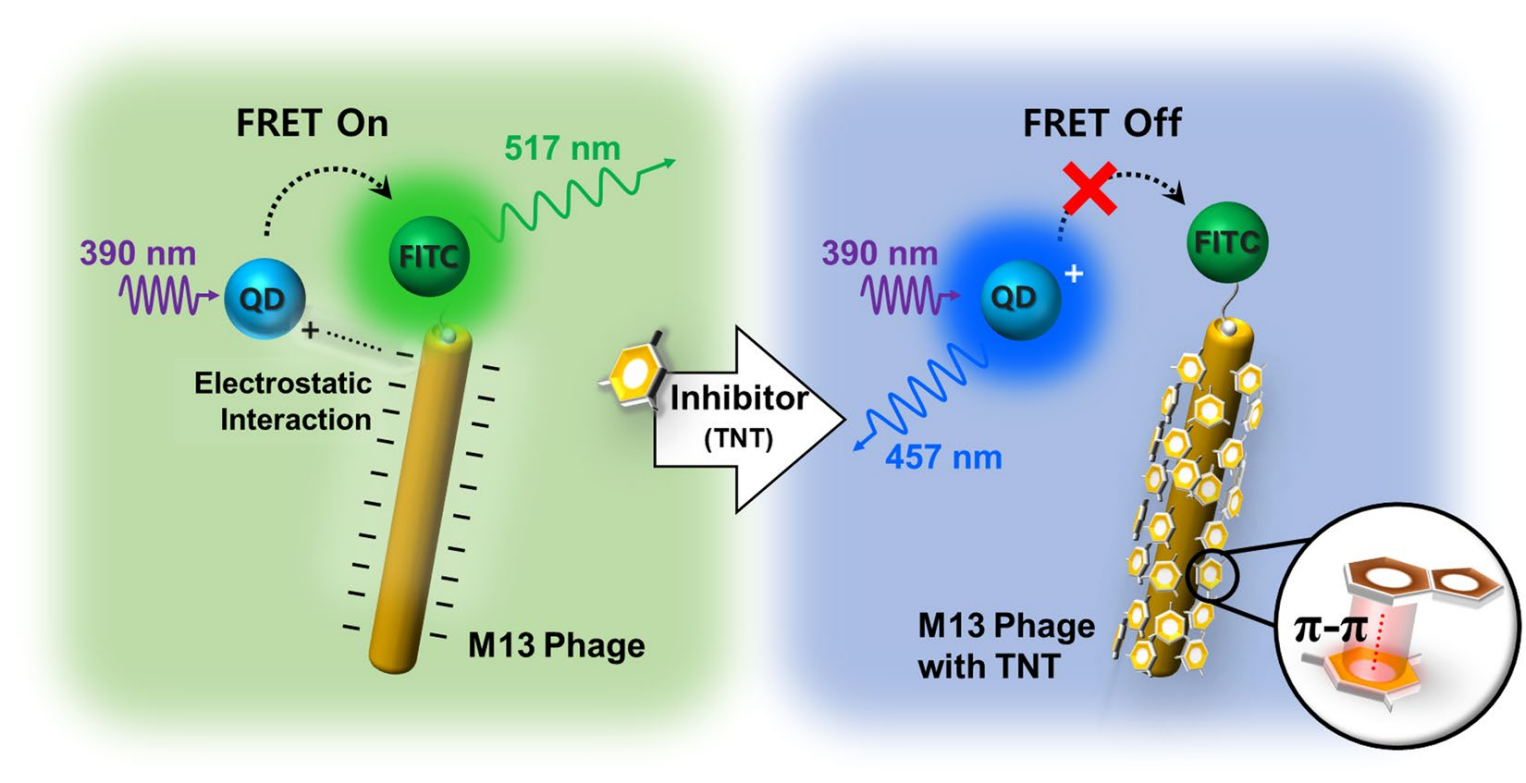
Concept illustration of a TNT blocking mechanism using an M13 bacteriophage-based FRET system.

Figure 1 illustrates a TNT blocking mechanism based FRET system using the genetically engineered M13 phage. To implement a resonance energy transfer system, water-soluble alloyed CdSSe/ZnS nanocrystal quantum dots (NQDs) and a fluorescein isothiocyanate-labeled M13 phage (FITC-M13 phage) were used as an energy donor and energy acceptor, respectively. NQDs were positively charged by a polydiallydimethyl-ammounium chloride (PDDA) organic coating layer and had no linkable functional groups. FITC was immobilized on the head of the M13 phage. For this, streptavidin-FITC and genetically engineered M13 phage with biotin-like peptides consisting of histidine (His, H), proline (Pro, P), and glutamine (Gln, Q) were used.

An M13 phage is negatively charged due to aspartic (Asp, A) and glutamic acids (Glu, E) that are regularly distributed on its surface, and it has been additionally genetically engineered to contain tryptophan (Trp, W) and His, which effectively bind aromatic compounds.

In a complex solution of NQDs and FITC-M13 phages, Coulomb interactions between the NQDs and M13 phages cause the particles to attract each other and provide an intermolecular distance suitable for efficient energy transfer from the NQDs to FITC. Therefore, FITC dominantly emits green fluorescence (515 nm) under UV light excitation (390 nm), as shown in Fig. 1, although it has a very low molar extinction coefficient at this wavelength (Fig. S1).

Suitable environments for FRET are significantly negatively affected in the presence of TNT. Under the same light excitation condition, the complex solution of NQD and FITC-M13 phage fluoresces blue (450 nm), indicating the hindering of energy migration. When TNT solution is added to the complex solution of NQD and FITC-M13 phage, TNT distrupts the electrostatic interactions between NQDs and the M13 phage. TNT binds to the amino acids of the peptides of the M13 phage due to noncovalent attraction between delocalized *π*-electrons distributed on their aromatic rings, called *π*-*π* interaction or *π*-stacking^61^. This noncovalent interaction between aromatic *π*-systems is caused by the quadrupole moment of the *π*-ring and substituent effects^49,62–64^. In this process, tryptophan having an electron-donating primary amino group readily reacts with an electron-withdrawing analyte, TNT, to form the Meisenheimer complex^65,66^.

**Figure 2:**
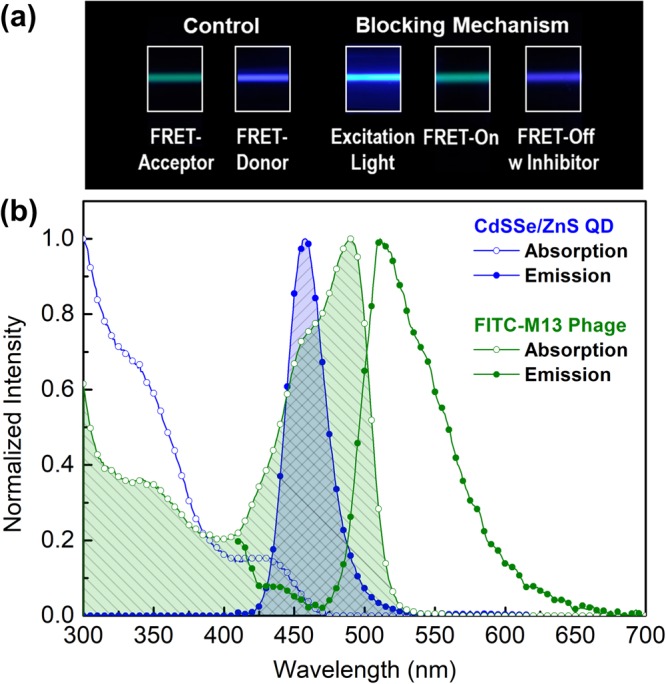
(**a**) The color change of separate and complex molecules in the absence and presence of NaCl. Cationic conjugated polymer and FITC-labelled M13 phage were used as the donor and acceptor, respectively. (**b**) The optical properties of CdSSe/ZnS QD (donor) and FITC (acceptor)-labeled M13 bacteriophage. Dotted line indicates Gaussian curve fitting of NQDs’ emission.

We first demonstrate a blocking mechanism to inhibit FRET sensing. To prove the mechanism, a FITC-conjugated M13-phage was prepared as a FRET-acceptor. As shown in Fig. 2(a), when the donor was added to the acceptor, energy transfer was observed without the presence of an inhibitor. The green light (FRET-on) is emitted by energy transfer from the FRET-donor, and we confirmed that the wavelength matched that of the FRET-acceptor. The blocking mechanism was experimentally confirmed with addition of the inhibitor. Under 400-nm laser excitation, the green emission of FITC was dominantly observed, while the emission wavelength was dramatically changed back to blue when the inhibitor was added to the solution. This phenomenon indicates that the energy transfer can be effectively controlled by the electrostatic binding of inhibitors.

For a better understanding of the FRET mechanism with M13 phages, the optical properties of CdSSe/ZnS NQDs (core-shell type) and the FITC-M13 phage were measured, as shown in Fig. 2(b). Under the irradiation of continuum light (Xe-lamp), the NQD ensemble exhibited a large interband absorbance, which depends on coupling between the valence and conduction bands of the CdSSe core in the UV region. In particular, two peaks (345 nm and 430 nm), which have relatively higher absorption coefficients than those of other spectral regions above the threshold wavelength (475 nm) for the carrier (electron) excitation, were observed. Since the absorption spectrum provides information about the electronic energy states within NQDs, we chose the suitable wavelength for carrier excitation at which photons can easily undergo an interband transition, allowing for these sub-bands^67^.

Under non-resonant excitation at a higher energy level (390 nm) than the band edge of NQDs, the emission spectra of NQDs showed a Gaussian-like shape with a normal distribution due to the size dispersion of the NQDs. The maximum fluorescence intensity of the NQDs was observed at 458 nm, and its FWHM (full width half maximum) was about 33 nm. At longer wavelengths than 580 nm, the fluorescence spectrum of the NQDs exhibited a distorted Gaussian symmetry, which indicated lower energy states within NQDs due to surface impurities or organic ligand materials that enable carrier trapping. Organic ligands used to synthesize the colloidal quantum dots or for surface passivation can act as fluorescence quenchers through molecular contact with excited carriers (self-quenching).

The FITC-M13 phage showed a broad absorption spectrum with a maximum intensity at 489 nm. Several sub-bands (350 nm and 462 nm) related to electronic vibrational energy states were also observed in the absorption spectrum. Unlike semiconductors, the energy states of organic molecules consist of electronic, rotational, and vibrational states grouped by spin multiplicity, because atoms can vibrate about their covalent bonds^67^. In addition, according to the Jablonski energy diagram^68^ of organic molecules, absorption occurs between the HOMO (highest occupied molecular orbital) and LUMO (lowest unoccupied molecular orbital). Nevertheless, light absorption by organic molecules should obey the selection rules. As a result, the energy bands observed in the absorption spectrum of the FITC-M13 phage indicated spin-allowed transitions, namely singlet-singlet or S_0_ → S_1_ transition^67^.

Unlike the fluorescence spectrum of donors, the FITC-M13 phage showed broad emission with a maximum peak at 511 nm. The optical transition between the HOMO and LUMO in organic dyes follows the Franck-Condon principle^68^. Thus, the emission spectrum of the FITC-M13 phage exhibited mirror symmetry in its absorption spectrum because the intensity of the vibronic transition is proportional to the overlap between the initial and final vibrational wave functions^67^. In addition, the spectral overlap between the donor emission spectrum and the acceptor absorption spectrum was analyzed, shown in Fig. 2(b). Spectral overlap (*J*) can be calculated by integrating the wavelength-dependent product of the normalized fluorescence intensity of the donor (*F*_D_(*λ*)) and the molar extinction coefficient of the acceptor (*ε*_A_(*λ*))^69^:

1 $$J=\int {F}_{{\rm{D}}}(\lambda ){\varepsilon }_{{\rm{A}}}(\lambda ){\lambda }^{4}d\lambda $$

This overlap provides a means of assessing the extent of resonant coupling between the donor and acceptor molecules and of determining the energy migration (Fig. S2).

**Figure 3:**
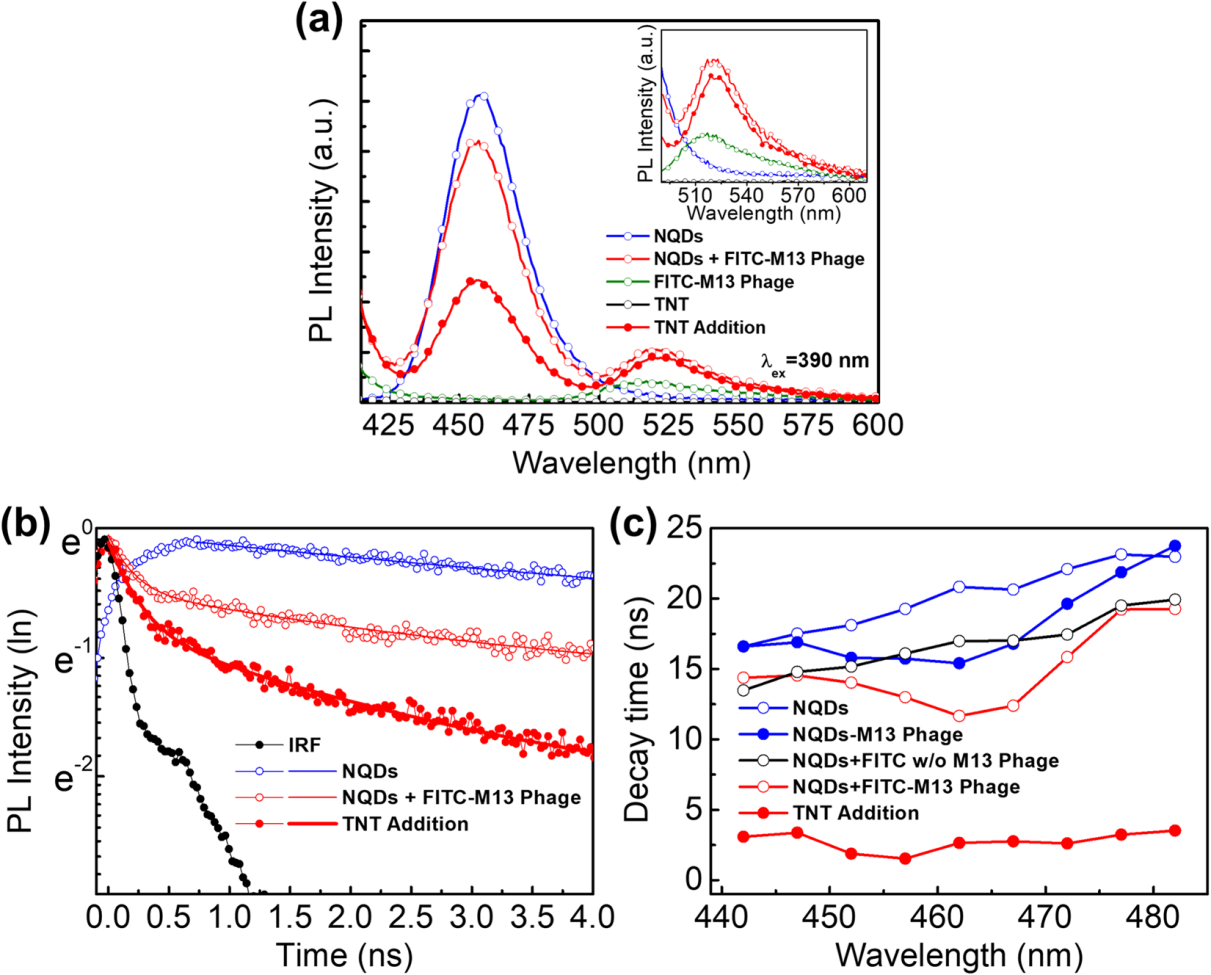
(**a**) The fluorescence intensities of separate molecules (donor and acceptor) and complex in the absence and presence of TNT were measured under 390-nm laser excitation. (**b**) The fluorescence decay dynamics of separate molecules and the complex donor in the absence and presence of TNT. Filled circles indicate the IRF signal with a FWHM of 220 ps. (**c**) The wavelength dependence of the donor fluorescence decay time in the absence and presence of TNT.

Figure 3 presents the fluorescence intensities of free NQD, the FITC-M13 phage and their mixture. Under 390-nm laser excitation, the fluorescence spectrum showed a significant change after interaction between the NQDs and FITC-M13 phages. Figure 3(a) is a fluorescence intensity spectrum of NQD and the FITC-M13 phage with NQD complex under 390-nm-wavelength light excitation. The intesity of the FITC-M13 phage should have relatively low emission due to the wavelegnth mismatch, as shown in the previous figure. However, the energy transfer between the NQDs and FITC-M13 phages attached by electrostatic interactions causes fluorescence emission at near 520 nm. Thus, it was proven that changes in the fluorescence intensities from the donor and acceptor originated from energy transfer between NQDs and FITC-M13 phages. On the other hands, in the presence of TNT, the fluorescence intensity of NQDs significantly decreased as shown in Fig. 3(a). This indicates that TNT can interact with NQDs regardless of the presence of FITC-M13 phage although NQDs have no functional group. Thus, we can expect that the total energy transfer is determined by the competition between TNT quenching effect on NQDs and FRET.

Fluorescence decay time was also measured to examine the FRET phenomenon between the NQDs and the FITC-M13 phages in Figs 3(b,c) and S3. Figure 3(b) shows the ultrafast decay dynamics of individual NQDs and the NQDs/FITC-M13 phage mixture. The fluorescence decay time is calculated by multi-exponential fitting as given below^70^,

2 $$I(t)={I}_{0}+{a}_{1}\,\exp (\,-\,t/{\tau }_{1})+{a}_{2}\,\exp (\,-\,t/{\tau }_{2})$$

where *a*_1_ and *a*_2_ are the weight factors of each of the decay components and *τ*_1_ and *τ*_2_ are the decay time. The average decay time is determined by

3 $$\langle \tau \rangle =({a}_{1}\,{\tau }_{1}+{a}_{2}\,{\tau }_{2})/({a}_{1}+{a}_{2})$$

Under excitation, excitons in a complex solution multi-exponentially decay, while they single-exponentially decay (8.54 ns) in a solution of only NQDs. On the other hand, the average lifetime of the excitons as calculated by the amplitude average^70^ of the decay components changed to 2.28 ns after complexation with FITC-M13 phages. These changes in the decay curve are phenomenologically reasonable because it has been reported that new decay pathways such as the energy transfer process lead to a reduction of the exciton lifetime^71–73^. Similarly, the fluorescence quenching effect by TNT as an additional decay process make decay time shorter than original decay time of NQDs.

The measurements of the fluorescence decay time of NQDs showed a wavelengh-dependent nature, shown in Fig. 3(c). In separate donor molecules, the fluorescence decay time increased from 16.6 ns to 22.97 ns as the detection wavelength increased. The fluorescence decay time of NQDs showed a parabolic shape in the presence of M13 phage, which has the shortest decay time (15.41 ns) at around 462 nm. In practice, resonant coupling between NQDs and FITC is the strongest at this wavelength. Interestingly, this tendency in the fluorescence decay time was also observed in the complex solution of NQDs and M13 phages without FITC. This phenomena represents that homo-FRET takes place within NQDs assembled to M13 phage by the electrostatic interaction^74^. On the other hands, this resonant coupling between NQDs becomes weak in the presence of TNT.

**Figure 4:**
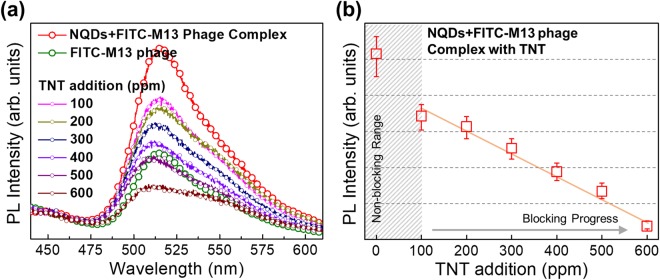
(**a**) Intensity spectra of the fluorescence dependence on the TNT concentration and the fluorescence of the NQDs, NQDs + FITC-M13 phage complex. After complexation of NQDs and FITC-M13 phage, the change in fluorescence intensity of NQDs is not distinguishable due to light scattering. (**b**) The reduced fluorescence intensities of FITC are indicated when TNT is added to the solution to act as a blocking material.

Finally, we applied the FRET system using the M13 phage to detect TNT concentrations. As we mentioned in Fig. 1, M13 phages are designed to strongly attract TNT since the Trp (W) and His (H) displayed on the surface of M13 phage have a high affinity for TNT^75^. Thus, noncovalent attractions occur between the amino acids of the peptides on M13 phages and the delocalized *π*-electrons distributed on the aromatic rings of TNT^49,62–64^. Since the change in fluorescence intensity of FITC-M13 phage by FRET mechanism corresponds to the reduction in that of NQDs, we first checked the TNT effect on NQDs (Figs S4–S6) when we added different concentrations of TNT in solution. And then added them to solutions of FITC-M13 phages. We expected the TNT to bind to the surface of M13 phages and interupt the FRET mechanisms. Figure 4(a) shows the FRET signal data from 100 ppm to 600 ppm TNT addition. In the presence of TNT, we found that the fluorescence intensities of FITC decreased. The fluorescence intensity of FITC decreased quite linearly and became saturated at concentrations above 600 ppm. The reduced fluorescence intensities of FITC which is attached on M13 phage are indicated in Fig. 4(b) by adding TNT in the solution to act as a suppressor. Eventually, the suppression of TNT in our FRET system could be determined by the binding affinity of phage. According to our previous results, M13 phage displayed with WHW peptides could detect TNT up to 300 ppb^49^. In case of FRET-based phage-anaylte system, the excellent sensitivity of 5ppb was reported^50^. However, our FRET system is more complicated because the interaction between NQDs and M13 phage was observed. Nevertheless, as indirectly guessed from the change in fluorescence intensity of FITC before and after complexation or TNT addition, the TNT suppressor ability of our system is about 400 ppm. In particular, it can be said that the blocking mechanism of TNT influences the relative intermolecular distance and suppresses the energy transfer from NQDs to FITC.

Here, we estimated the relative intermolecular distance between the donor and acceptor based on the energy transfer efficiency. The relative donor-to-acceptor intermolecular distance (*R*_DA_) can be calculated by the Förster theory^68^ when the Förster distance (*R*_0_), which is the distance at 50% efficiency of energy transfer, is 53.21 Å:

4 $${E}_{{\rm{FRET}}}={R}_{0}^{6}/({R}_{0}^{6}+{R}_{{\rm{DA}}}^{6})$$

The theoretical FRET efficiency equation, which is only determined by the FRET term, could not be applied on a practical FRET system because of fluorescence quenching. Various exciton deactivation processes such as charge transfer or carrier trapping including self-quenching drive fluorescence quenching^76–79^. Moreover, the presence of charged fluorescence quenchers can lead to the transformation of the overall dipole distribution. Thus, random dipole orientation factors^80^ used generally in liquid solution may no longer be valid.

**Figure 5:**
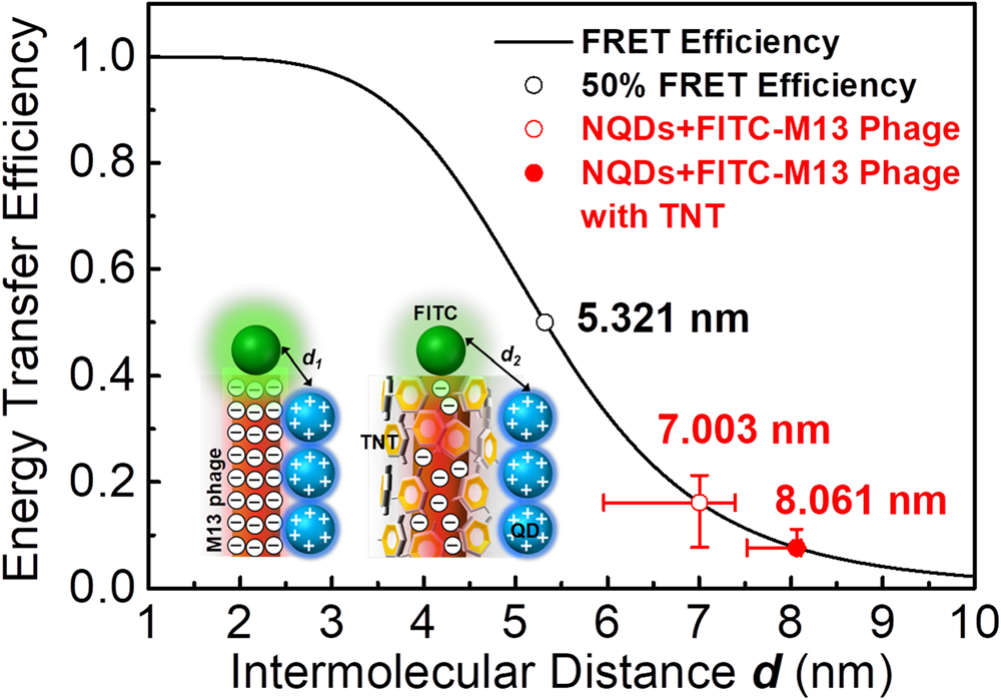
The energy transfer efficiency and the relative intermolecular distance in the absence and presence of TNT.

Nevertheless, we can expect that the energy transfer efficiency is inversely proportional to ${R}_{{\rm{DA}}}^{6}$ because the efficiency is dominantly decided by the energy transfer between the donor and acceptor. Considering the morphology of constituents within our FRET system, NQDs and M13 bacteriophage may be aggregated each other or single NQDs may be bound on the surface of M13 bacteriophage and arranged along to its surface by Coulomb interaction. In addition, the radius of NQDs is more two times longer than the distance between subunits (N-termini) of M13 phage and the hopping or the energy transfer of excited carriers on the surface of M13 phage will be randomly progressed. Thus, the relative intermolecular distances between NQDs and FITC-M13 phages can be calculated by the effective energy transfer efficiency. Figure 5 shows the relative intermolecular distances between NQDs and FITC-M13 phages. In the absence of TNT, the donor-to-acceptor distance was estimated to be 70.03 Å, and this value is reasonable considering the sizes of alloyed CdS_x_Se_x−1_ NQDs^81^ and FITC. On the other hand, in the presence of TNT, the relative donor-to-acceptor intermolecular distance was changed to 80.61 Å.

## Discussion

Here, to more quantitatively analyze the temporal dynamics of donors in a complex solution of NQDs and FITC-M13 phages, we applied the rate equation involved in exciton decay. In general, the fluorescence decay rate of the donor (*k*_F_) is defined by ${k}_{{\rm{F}}}={k}_{{\rm{rad}}}+{k}_{{\rm{nonrad}}}$, where *k*_rad_ and ${k}_{{\rm{F}}}={k}_{{\rm{rad}}}+{k}_{{\rm{nonrad}}}$ are the radiative and nonradiative decay components, respectively^68^. Since ${k}_{{\rm{nonrad}}}$ is associated with intrinsic properties of a separate donor molecule, such as trap sites or impurities, we can rewrite as *k*_int_. If a new decay process (*k*_new_) producing a reduction in the fluorescence intensity is present, the previous decay rate equation should be modified as

5 $${k}_{{\rm{F}}}^{\ast }={k}_{{\rm{rad}}}+{k}_{{\rm{int}}}+{k}_{{\rm{nonrad}}}^{\ast }\pm {k}_{{\rm{new}}}$$

where ${k}_{{\rm{nonrad}}}^{\ast }$ is an additional nonradiative component. However, ${k}_{{\rm{nonrad}}}^{\ast }$ is negligible because the discrepancy in the fluorescence intensity of NQDs before and after the complexation with M13 phage is attributed to a change in the concentration of NQDs (Fig. S7). Thus, the fluorescence decay rate of the donor in a complex solution of NQDs and FITC-M13 phages in which FRET is occurring can be rewritten as

6 $${k}_{{\rm{F}}}^{\ast }={k}_{{\rm{rad}}}+{k}_{{\rm{int}}}+{k}_{{\rm{FRET}}}$$

where ${k}_{{\rm{FRET}}}$ has a positive value because the fluorescence intensity of the donor is quenched by FRET.

Assuming that FRET is only involved in the fluorescence quenching of the donor, the energy transfer efficiency (${E}_{{\rm{FRET}}}$) can be theoretically calculated by

7 $${E}_{{\rm{FRET}}}=\frac{{k}_{{\rm{FRET}}}}{{k}_{{\rm{rad}}}+{k}_{{\rm{int}}}+{k}_{{\rm{FRET}}}}=\frac{{I}_{{\rm{D}}}-{I}_{{\rm{DA}}}}{{I}_{{\rm{D}}}}$$

where *I*_D_ and *I*_DA_ are the fluorescence intensities in the absence and presence of an acceptor, respectively^60^. In the complex solution of NQDs and FITC-M13 phage, the calculated FRET efficiency of the donor was 16.1%.

However, in the presnece of TNT, the previous rate equation for the donor is no longer valid because TNT produces additional quenching of the fluorescence intensity of the donor. Thus, the fluorescence decay rate of the donor including a new carrier deactivation process due to TNT should be corrected as

8 $${k}_{F-\mathrm{TNT}}^{\ast }={k}_{{\rm{rad}}}+{k}_{{\rm{int}}}+{k}_{{\rm{FRET}}}+{k}_{{\rm{TNT}}}$$

where *k*_TNT_ is the exciton decay rate due to TNT. Therefore, we can calculate the effective energy transfer efficiency (*E*_eff_) in the presence of TNT as given below.

9 $${E}_{{\rm{eff}}}=\frac{{k}_{{\rm{FRET}}}}{{k}_{{\rm{rad}}}+{k}_{{\rm{int}}}+{k}_{{\rm{FRET}}}+{k}_{{\rm{TNT}}}}=\frac{{I}_{{\rm{D}}}-{I}_{{\rm{DA}}-{\rm{TNT}}}}{{I}_{{\rm{D}}}}$$

where ${I}_{\mathrm{DA}-\mathrm{TNT}}$ is the fluorescence intensity of the donor in the presence of TNT. The subscript DA indicates a complex solution of NQDs and FITC-M13 phages (Fig. S8). The effective energy transfer efficiency in the presence of TNT was 7.6%, while the total fluorescence quenching efficiency including FRET and the TNT-related effect was 60.4% (S9). In particular, this quantitative estimation for M13 phage-TNT interaction provides the scalability to other biomarker detection since M13 phage can be specified for analyte detection by phage display.

## Concluding Remarks

We demonstrated the effective discrimination of TNT using an M13 phage-based on/off FRET sensor. In on/off-mode operation, our sensor sensitively detected incoming TNT through a spectral change from green to blue color due to FRET on/off switching. This feature was also confirmed from the change in the fluorescence intensity and temporal behavior of exciton decay. During on/off switching of the FRET due to TNT, the total energy transfer efficiency and the relative donor-to-acceptor intermolecular distance were effectively controlled by the change in the electrostatic interaction between NQDs and M13 phages. By phenomenological analysis, the energy transfer efficiency and relative intermolecular distance were also estimated. In particular, our M13 phage-based FRET system followed the original distance dependence of (1/R_DA_)^6^, although the total fluorescence quenching efficiency was controlled by the combined process of energy harvesting and carrier deactivation. Consequently, this on/off sensing shows that M13 phage not only directly controls the energy transfer but also simultaneously detects incoming analytes through the bimolecular FRET system. In our study, a fascinating biomaterial, M13 phage, was utilized as an independent functional system for FRET on/off switching using thier unique protein display platform.

## Experimental Section

### Sample preparation

As energy donors for FRET application, water-soluble alloy CdSSe/ZnS core/shell quantum dots with an amphiphilic polymer and PDDA coating (QSQ-450, Ocean Nano Tech) were used. PDDA was used for good aqueous solubility as well as electricity (positive charge). While oleic acid or octadecylamine, which used in the synthesis process of inorganic core-shell quantum dots, are insoluble in water, NQDs can become soluble in water by the wrapping of amphiphilic polymer and PDDA. The concentration of the stock solution was 8 μΜ. There was no linkable reactive group on their surface. The quantum yield of NQDs was above 50%. Streptavidine-FITC conjugate (Sigma-Aldrich) was used as the energy acceptor. Lyophilized powder was dissolved in distilled water (DI) at 1 mg/ml concentration. The extent of labeling was 3~9 mol FITC per mol streptavidine. For complexation of the donor and acceptor, a 1/20 diluted NQD solution was used because of its high molar extinction coefficient compared to FITC, while a stock solution of FITC was used as an acceptor. After mixing of the NQDs (4 nM) and FITC (2.7 nM) in DI water, the time-integrated and time-resolved fluorescence were measured. TNT powder was dissolved in a mixture of dimethyl sulfoxide (DMSO) and DI water (DMSO:DI water = 2:1). For TNT detection, a 100 ppm solution was repeatedly added to the complex solution of NQDs and FITC-M13 phage.

### Genetic engineering of the M13 phage

For the selective detection of TNT, genetically engineered M13 phage in which Trp (W) and His (H) were displayed on their surface was used (WHW-phage). The detailed processes for phage synthesis and amplification are based on the previous reports^49^. To make sure the phages are purified before use in FRET system, the WHW phages DNA were purified by DNA isolation kit and the sequence analysis. The detailed information of the phage purification process is described in S10 and fig. S10. The concentrations of the phage suspensions were calculated by the formula given below:

$${\rm{mg}}\,{\rm{of}}\,{\rm{phages}}/{\rm{mL}}=({\rm{A}}269-{\rm{A}}320)/3.84$$

where A269 and A320 represent the absorbance of the phage suspension at 269 nm at 320 nm, respectively. In all measurements, 5 mg/ml M13 phage stock solution was used with additional dilution in DI water.

### Time-integrated Fluorescence measurement

The fluorescence intensities of NQDs and FITC were measured by using a 390-nm pulsed diode laser (N-390, NanoLED, Horiba Jobin Yvon) and a spectrofluorometer (Fluorolog-3, Horiba Jobin Yvon). Under laser excitation, the fluorescence signal was recorded using a photomultiplier tube (PMT, R928P, Hamamatsu Photonics). The bandwidth and light intensity of detected signals were controlled by slits in the monochromators. The spectral resolution was about 0.5 nm. All measurements were carried out at room temperature.

### Time-resolved Fluorescence measurement

The ultrafast decay dynamics of NQDs and FITC were measured using a time-correlated single photon counting (TCSPC) system (SPC-130EM, Becker & Hickl GmbH). As an excitation light source, a 404-nm pulsed diode laser (LDH series, PicoQuant) was used. The repetition rate of the pulse was adjusted by a PDL 800-B controller (PicoQuant). The pulse duration of the laser was below 75 ps. The fluorescence signal was collected by a PMT detector (PMH-100, Becker & Hickl GmbH). The time resolution was below 220 ps. All measurements were carried out at room temperature.

## Supplementary information

Dataset
