# Supplementary material for: Intermolecular distance measurement with TNT suppressor on the M13 bacteriophage-based Förster resonance energy transfer system
Source: Sci Rep. 2019 Jan 24;9:496. doi: 10.1038/s41598-018-36990-0 (PMC6345812; doi:10.1038/s41598-018-36990-0)

Supporting Information

# Intermolecular distance measurement with TNT suppressor on the M13 bacteriophage-based Förster resonance energy transfer system

Inhong Kim[a,†], Hyerin Song[b,†], Chuntae Kim[c,d], Minwoo Kim[b], Kwangseuk Kyhm[b], Kyujung Kim[b]*, and Jin-Woo Oh[c,d,]*

[a]School of Electrical Engineering and Computer Science, Gwangju Institute of Science and Technology, Gwangju, 61005, Republic of Korea
[b]Department of Cogno-Mechatronics Engineering, Pusan National University, Busan, 46241, Republic of Korea
[c]Department of Nano Fusion Technology, Pusan National University, Busan, 46241, Republic of Korea
[d]BK21 PLUS Division of Nano Convergence Technology, Pusan National University, Busan, 46241, Republic of Korea
[†]These authors contributed equally to this work.
*Corresponding E-mail: k.kim@pusan.ac.kr (K. Kim), ojw@pusan.ac.kr (J.-W. Oh)

**Fig. S1.** Fluorescence intensity of FITC under light excitation with different wavelength.

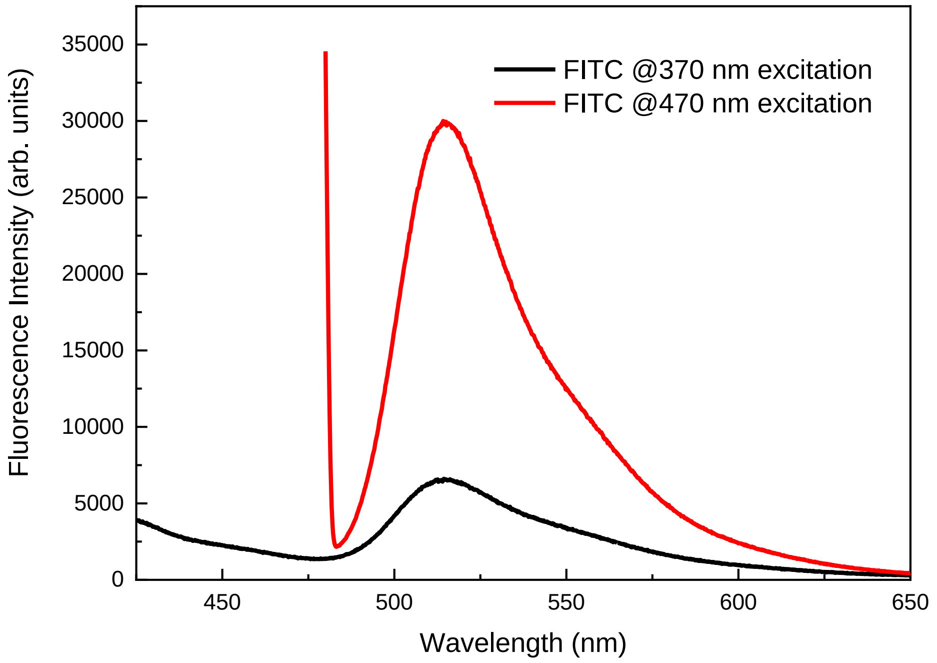

**Fig. S2.** Spectral overlap between NQDs and FITC-M13 phage.

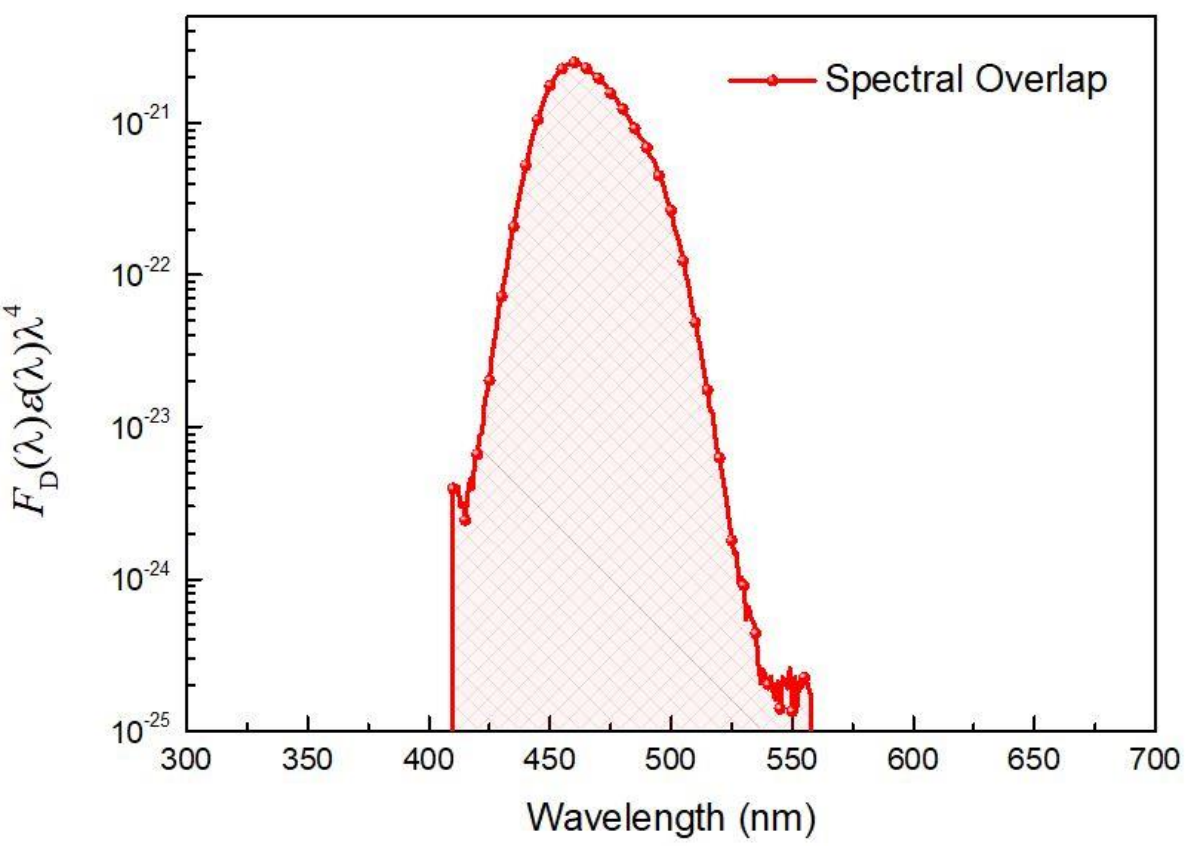

**Fig. S3.** (a) Fluorescence decay time of FITC in the absence and presence of TNT. (b) Wavelength dependence of fluorescence decay time of FITC in the absence and presence of TNT. The fluorescence decay time is calculated by multi-exponential fitting as given below $I(t) = I_0 + a_1 \exp(-t/\tau_1) + a_2 \exp(-t/\tau_2)$ where $a_1$ and $a_2$ are the weight factors of each of the decay components and $\tau_1$ and $\tau_2$ are the decay time. The average decay time is determined by $\langle\tau\rangle = (a_1 \tau_1 + a_2 \tau_2)/(a_1 + a_2)$.

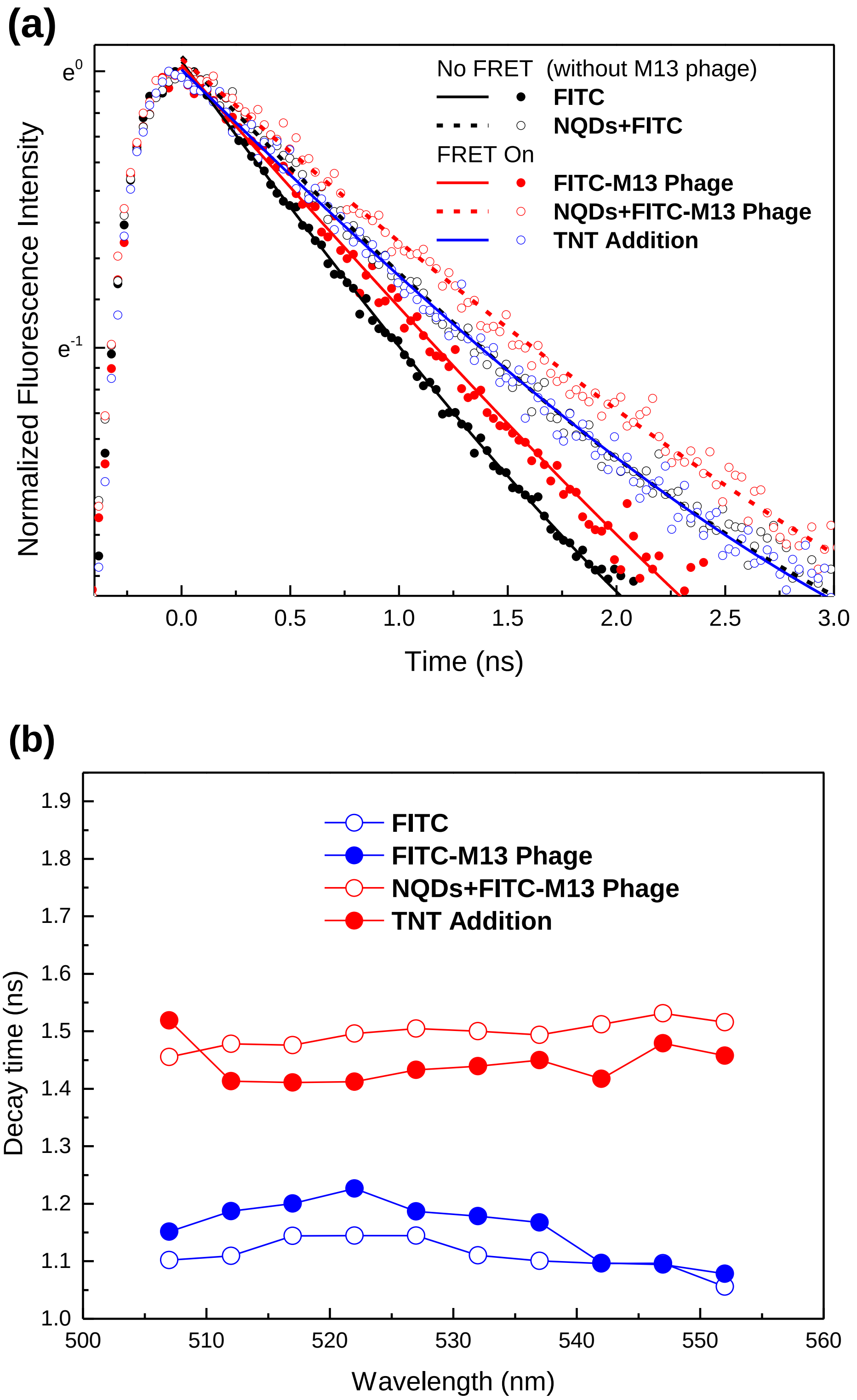

**Fig**. **S4**. Fluorescence intensity of NQDs without FITC-M13 Phage with different concentration in the absence and presence of TNT.

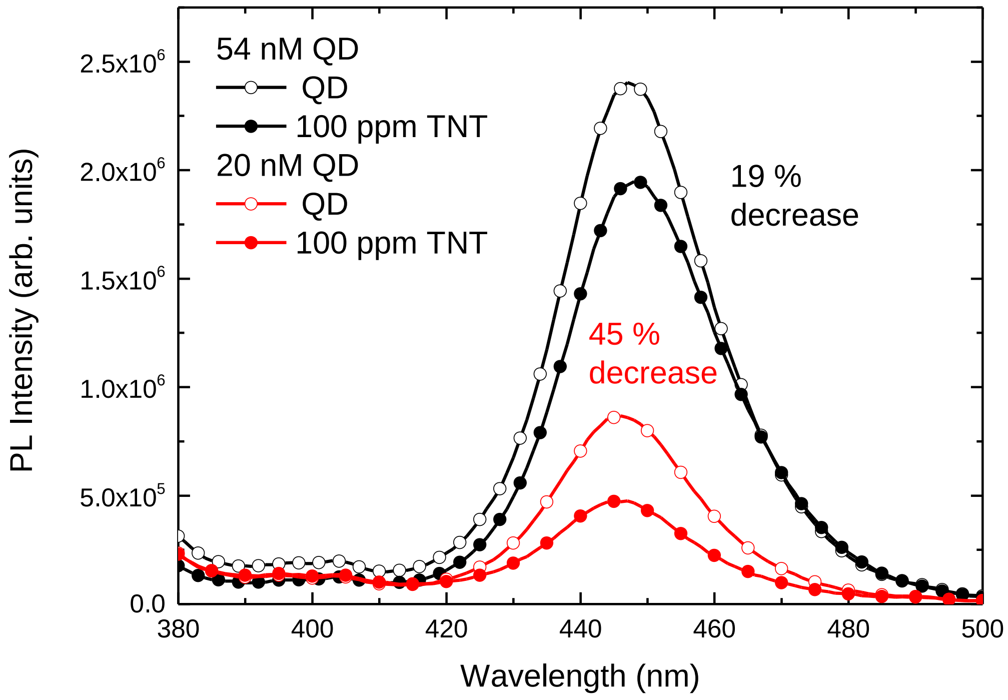

**Fig**. **S5**. Fluorescence intensity of NQDs in the absence and presence of TNT.

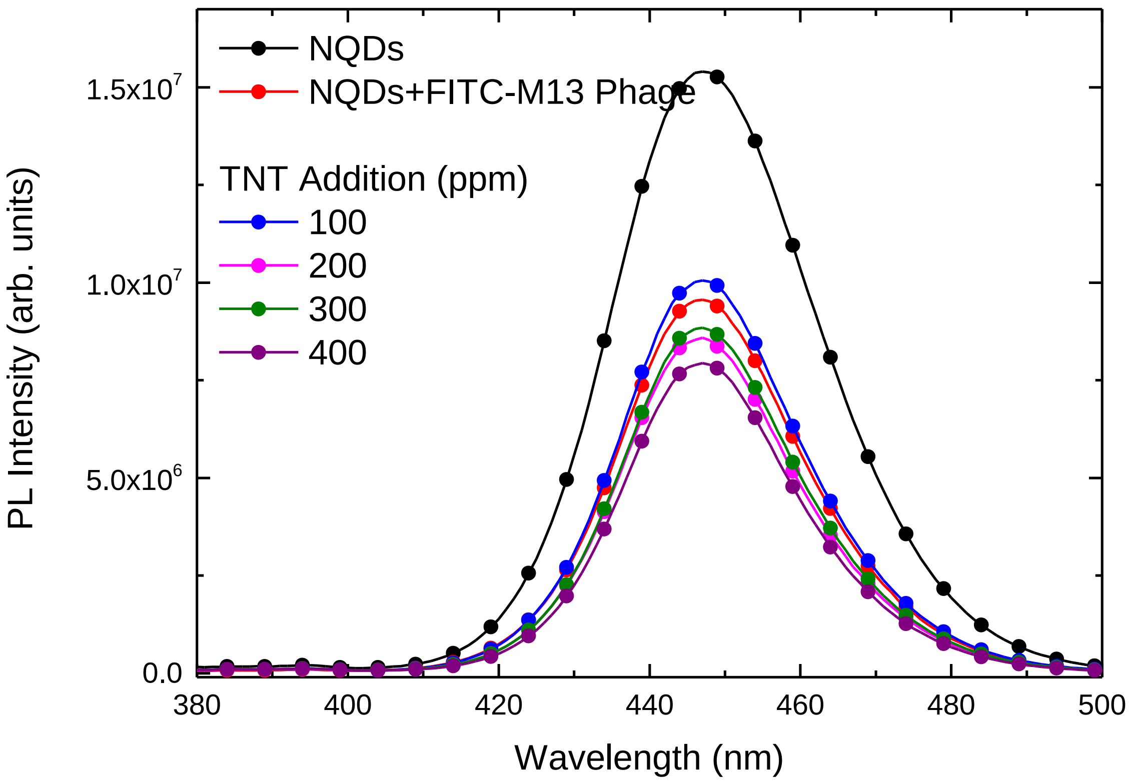

**Fig**. **S6**. Attenuation ratio of fluorescence intensity of NQDs in the absence and presence of TNT.

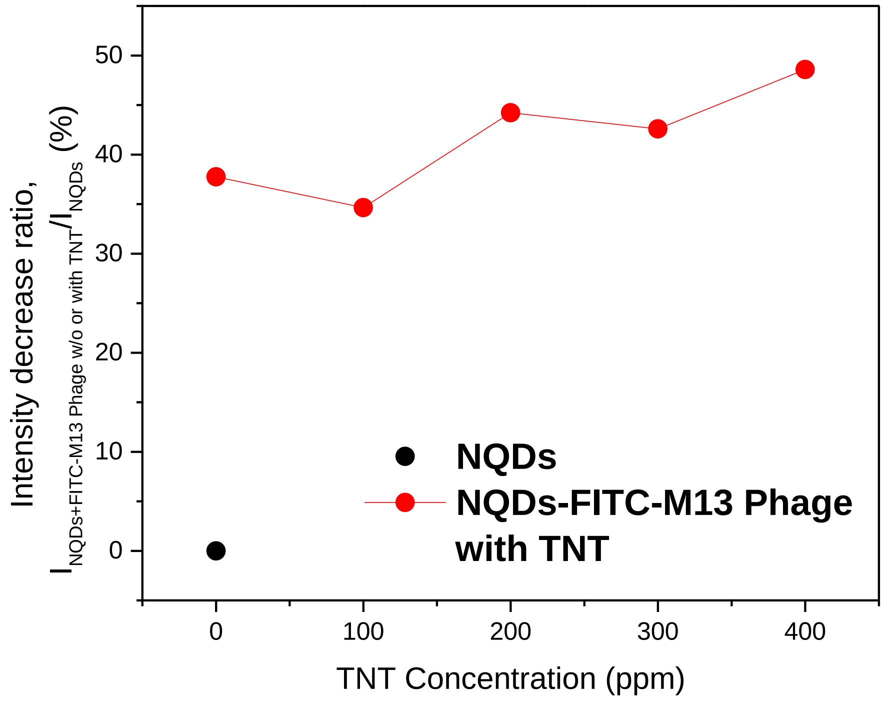

**Fig. S7.** (a) The fluorescence intensity of separate NQDs and the NQDs/M13 phage mixture. (b) The fluorescence intensity of separate FITC and the FITC-M13 phage. The difference in the fluorescence intensity between (a) and (b) was caused by the difference in the concentration of the solution due to the addition of M13 phage.

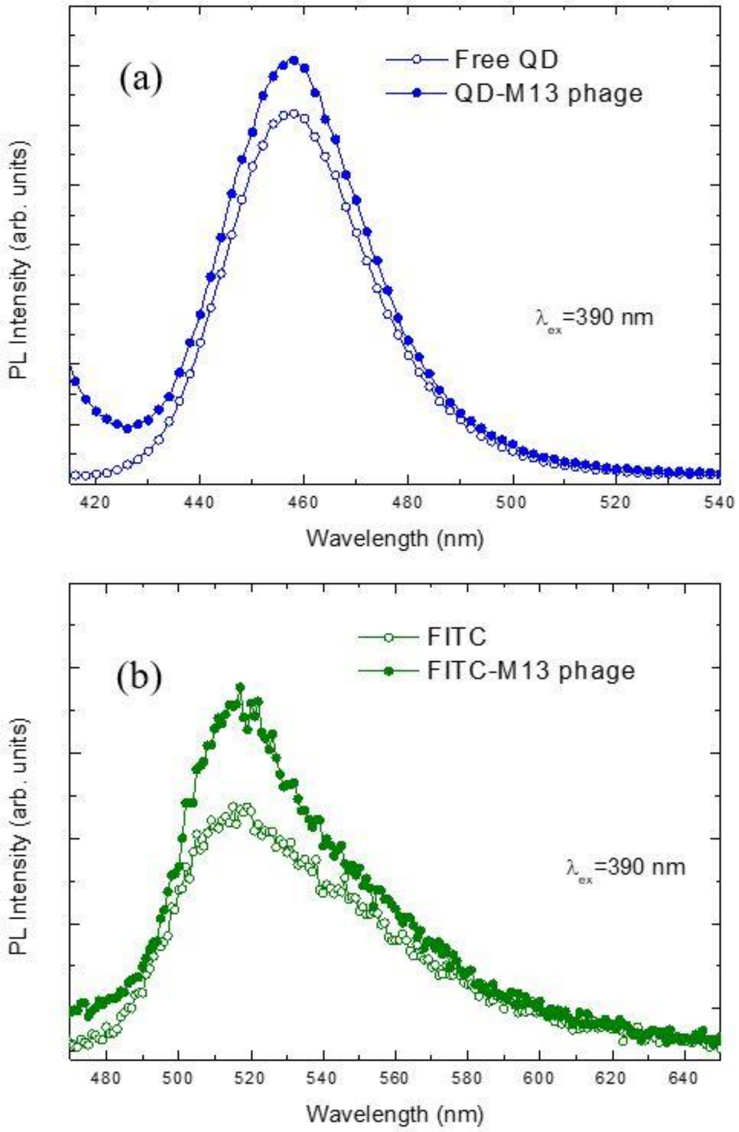

**Fig. S8.** Wavelength dependent energy transfer efficiency of NQDs+FITC-M13 phage complex in the absence and presence of TNT, respectively.

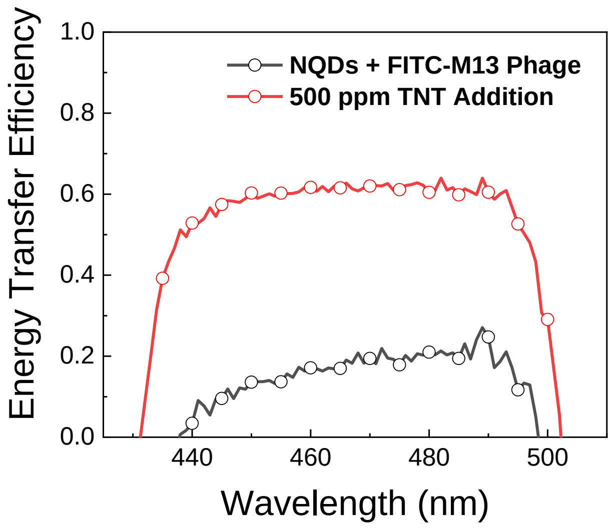

**S9.** Rate equation and energy transfer efficiency calculation

In general, the fluorescence decay rate is determined by the sum of each decay component. Thus, the rate equations for fluorescence decay dynamics in the NQDs-FITC-M13 phage and NQDs-FITC-M13 phage-TNT systems are given below.

$$k_{\mathrm{F}} = k_{\mathrm{rad}} + k_{\mathrm{nonrad}}$$

$$k_{\mathrm{nonrad}} = k_{\mathrm{int}}$$

The fluorescence decay rate ($k_{\mathrm{F}}$) is generally defined by $k_{\mathrm{F}} = k_{\mathrm{rad}} + k_{\mathrm{nonrad}}$, where $k_{\mathrm{rad}}$ and $k_{\mathrm{nonrad}}$ are the radiative and nonradiative components, respectively. Since $k_{\mathrm{nonrad}}$ is involved in the intrinsic properties of a separate donor, it can be denoted by $k_{\mathrm{int}}$.

As before, the fluorescence decay rate after complexation ($k_{\mathrm{F}}^{*}$) including the term for energy transfer can be defined as

$$k_{\mathrm{F}}^{*} = k_{\mathrm{rad}} + k_{\mathrm{nonrad}}^{*}$$

$$k_{\mathrm{nonrad}}^{*} = k_{\mathrm{int}} + k_{\mathrm{FRET}},$$

where $k_{\mathrm{FRET}}$ is the FRET decay rate.

In the presence of TNT, the total decay rate ($k_{\mathrm{F\text{-}TNT}}^{*}$) is defined as

$$k_{\mathrm{F\text{-}TNT}}^{*} = k_{\mathrm{rad}} + k_{\mathrm{nonrad\text{-}TNT}}^{*}$$

$$k_{\mathrm{nonrad\text{-}TNT}}^{*} = k_{\mathrm{int}} + k_{\mathrm{FRET}} + k_{\mathrm{TNT}},$$

where $k_{\mathrm{nonrad\text{-}TNT}}^{*}$ is the nonradiative decay rate in the NQDs-FITC-M13 phage-TNT system and $k_{\mathrm{TNT}}$ is the nonradiative decay rate of TNT particles.

The efficiency is determined by the ratio of each decay rate to the whole decay rate. Thus, the energy transfer efficiency ($E_{\mathrm{FRET}}$) can be obtained from the rate equation. The calculated energy transfer efficiency, quenching efficiency of TNT ($E_{\mathrm{TNT}}$), and effective energy transfer efficiency ($E_{\mathrm{eff\text{-}TNT}}$) are given below.

$$E_{\mathrm{FRET}} = \frac{k_{\mathrm{FRET}}}{k_{\mathrm{rad}} + k_{\mathrm{int}} + k_{\mathrm{FRET}}} = 0.161$$

$$E_{\mathrm{FRET\text{-}TNT}} = \frac{k_{\mathrm{FRET}} + k_{\mathrm{TNT}}}{k_{\mathrm{rad}} + k_{\mathrm{int}} + k_{\mathrm{FRET}} + k_{\mathrm{TNT}}} = 0.604$$

$$E_{\mathrm{TNT}} = \frac{k_{\mathrm{TNT}}}{k_{\mathrm{rad}} + k_{\mathrm{int}} + k_{\mathrm{FRET}} + k_{\mathrm{TNT}}} = 0.528$$

$$E_{\mathrm{eff\text{-}TNT}} = \frac{k_{\mathrm{FRET}}}{k_{\mathrm{rad}} + k_{\mathrm{int}} + k_{\mathrm{FRET}} + k_{\mathrm{TNT}}} = 0.076$$

**S10.** Phage purification process

**Generation of phage DNA**

To generate functional peptide on phage surface, we adapted the inverse PCR method. Each primer was designed to suit application. The pVIII Forward primer was designed to include the Pst I restriction site, the insert sequence, 5'-ATATATCTGCAGNKNNKTGGCATTGGCAGNNKNNKGATCCCGCAAAAGCGGCCTTTAACTCCC -3'. And the reverse primer was designed to make the vector linear and complimentary to the engineered gVIII 3'-5' region, 5'-GCTGTCTTTCGCTGCAGAGGGTG – 3'.

We analyze the base sequence using -96 primer (5'- GCC CTC ATA GTT AGC GTA ACG-3') through the reverse direction. And we got each sequence of VIII peptide (Wild type & WHW type). After searching the start codon of pVIII and aligning to base information, we confirm the peptide sequences.

**Extraction of phage DNA and sequencing**

DNA was extracted using DNA Plasmid Isolation kit (Bionner. Co. Korea) and was sent to BIONICS (Bionics Co., Ltd, KOREA) for DNA sequencing and alignment of the amino acid sequences.

**DNA sequencing for WHW phage**

The WHW phages DNA were purified by DNA isolation kit and the sequence analysis. The results of pVIII Wild and pVIII WHW gene coding base pair sequences are illustrated Fig. S11 (a), the WHW sequencing analysis has 85% identify with protein M13 f1 pVIII when compared with the Basic Local Alignment Search Tool (http://www.ncbi.nlm.nih.gov/Blast) in Fig S11 (b). The amino acids translated by DNA sequences are presented, in which a sequence (AAEE**WHW**QEGD) appeared thrice; this WHW sequence is analyze with the Chromas (Version 2.6.4) Tool (Technelysium Pty. Ltd). The DNA sequencing of WHW phage 3 peptide fragments is illustrated in Fig. S11 (c).

**Fig. S11.** DNA sequencing and analyze of the WHW phage clone. (a) pVIII and pVIII WHW gene coding base pair sequences. (b) pVIII WHW amino acid frequency and identify analyze with pVIII Wild and (c) DNA sequencing of pVIII Wild (left) *vs* pVIII WHW (right). Under bar indicating the WHW peptide insertion sites only pVIII WHW sequence.

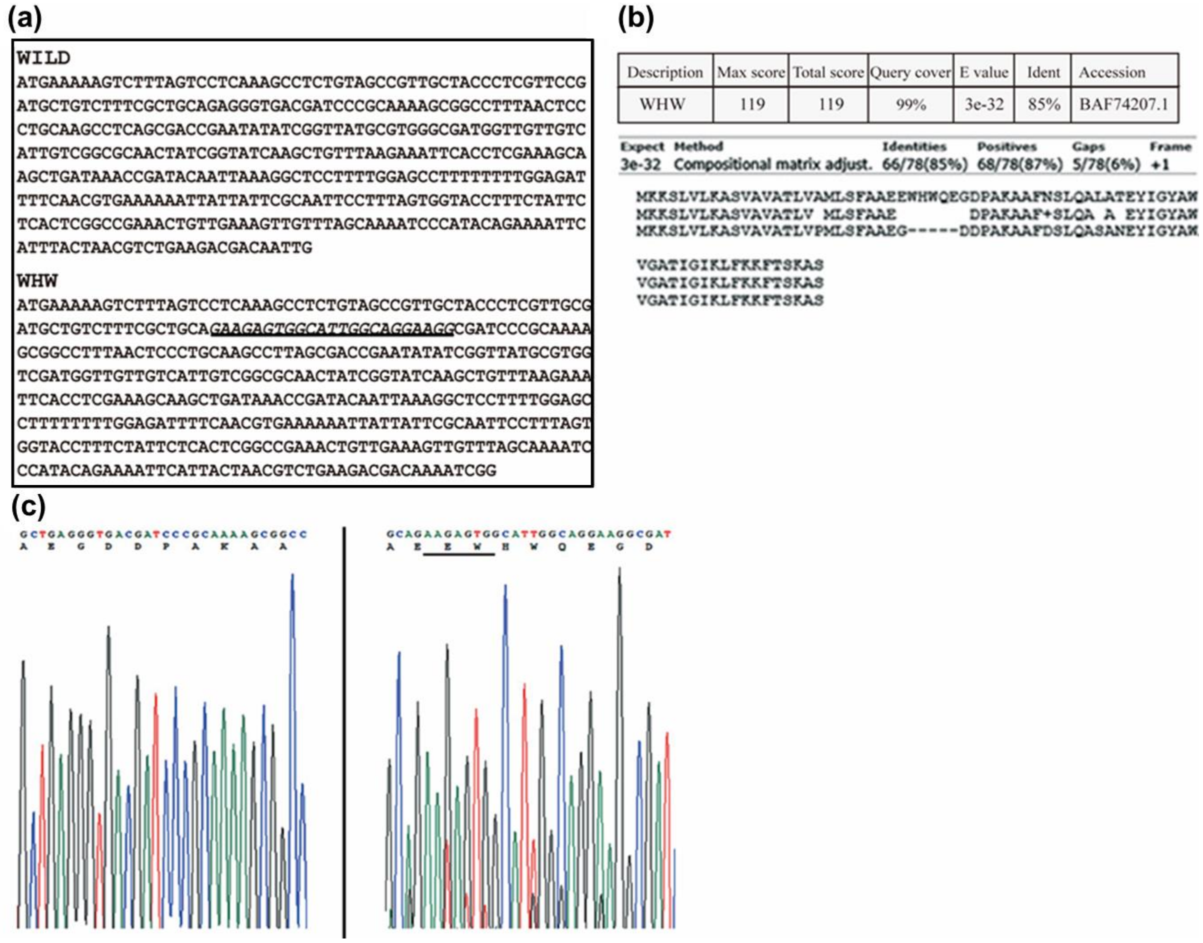

Supplement: Supplementary file 1 — Dataset [file 41598_2018_36990_MOESM1_ESM.pdf]
